# Cyclin D1 Again Caught in the Act: Dyrk1a Links G1 and Neurogenesis in Down Syndrome

**DOI:** 10.1016/j.ebiom.2015.02.003

**Published:** 2015-02-07

**Authors:** Ian Smith, Federico Calegari

**Affiliations:** DFG—Research Center and Cluster of Excellence for Regenerative Therapies, TU-Dresden, Fetscherstrasse 105, 01307 Dresden, Germany

Down syndrome (DS) was first described in 1866 by John Langdon Down and it is now recognized as the most common chromosomal disorder and cause of intellectual disability (Haydar and Reeves, 2012). Despite its prevalence (roughly 1 in 1000 births), the cause of the disease was not identified until 1959 when karyotyping enabled the identification of trisomy 21 as responsible for the majority of DS cases (Haydar and Reeves, 2012). The complete sequence of chromosome 21 was determined in the year 2000 (Hattori et al., 2000). Yet, as researchers have discovered time and again, knowledge of an organism's genetic sequence does not necessarily translate into a precise understanding of molecular and cellular processes. Almost sixty years have passed since the cause of the disease was identified; yet the mechanisms underlying DS remain obscure.

In this issue of *EBioMedicine*, Najas et al. expand our knowledge of DS by showing that the dosage of the evolutionarily conserved tyrosine kinase Dyrk1a, encoded by a gene located on chromosome 21 and triplicated in DS, alters the cell cycle, and hence the fate, of neural stem cells (Najas et al., 2015-in this issue).

During mammalian corticogenesis, radial glial (RG) cells primarily divide to either expand their own population or produce intermediate progenitors (IPs). IPs have limited proliferative potential and predominantly generate neurons (Taverna et al., 2014). Interestingly, both the regulation of the switch of RG cells from expansion to generation of IPs as well as the proliferative potential of IPs are intimately linked to cell cycle length (Borrell and Calegari, 2014). Specifically, the G1 phase is known to lengthen as neurogenesis progresses and overexpression of cell cycle regulators such as cyclin D1 shortens G1 and promotes proliferative (at the expense of neurogenic) divisions (Borrell and Calegari, 2014).

Changes in cell cycle regulation and cell fate change of neural progenitors were already reported in trisomic mouse models of DS (Haydar and Reeves, 2012) and it was also known that Dyrk1a could somehow influence both cell cycle progression and neurogenesis in vitro and in vivo (Soppa et al., 2014, Yabut et al., 2010). However, these previous studies could not assess the dose-dependent effect of Dyrk1a and quantify its effects on specific phases of the cell cycle. To address this, Najas and colleagues generated a number of mouse lines in which either three or one allele of Dyrk1a was present. Focusing on the developing brain, they observed decreased nuclear levels of cyclin D1 in the mouse line with three alleles while, conversely, finding more in the single-allele, haploinsufficient Dyrk1a line. Explaining this phenotype, Dyrk1a was found to phosphorylate cyclin D1 in vitro and likely disrupt its trafficking and degradation. Consistent with a decrease in the levels of cyclin D1, the authors found that an increase in the Dyrk1a gene dosage caused a lengthening of both G1 and S phases. This correlated with a transient increase in IPs followed by their premature depletion, which resulted in a transient increase in neurons at mid-neurogenesis followed by an overall reduction in the total neuronal output of the postnatal brain. Haploinsufficient mice displayed a converse phenotype, shedding light on the mechanistic link between gene-dosage of Dyrk1a and developmental malformations associated with DS.

To explain the parallel effect of Dyrk1a on the cell cycle and fate of neural progenitors, the authors refer to previous reports showing that lengthening G1 by decreasing the activity of cyclin D1 induces the premature generation of neurons and depletion of the stem cell pool (Borrell and Calegari, 2014). This is indeed consistent with observations by Najas et al. at mid/late-corticogenesis. Yet it is puzzling that more IPs and less neurons were also observed during early corticogenesis, which is the opposite of what one would expect from a manipulation that lengthens G1 (Borrell and Calegari, 2014). The authors propose that this might be explained by intrinsic differences in progenitor cells and/or environmental cues present at early versus late stages of corticogenesis. Yet, this does not entirely solve the issue since reports have shown that lengthening G1 results in a transient increase in neurons both at the onset (Calegari and Huttner, 2003) as well as at mid-neurogenesis (Lange et al., 2009). Perhaps, instead of differences in progenitors and/or cues within the niche, this discrepancy might be explained by the fact that previous studies were performed acutely and, often, tissue-specifically. While Najas et al. obtained an exquisite control of gene dosage, their manipulations were neither temporally nor spatially controlled among tissues physiologically expressing Dyrk1a.

Nevertheless, the powerful animal models generated and characterized by Najas et al. will be extremely useful in future research. They open up a number of possibilities, not only to study the pathology of DS during development, but also to investigate the role of Dyrk1a in the neurophysiology of postnatal neurons as these are known to display reduced synaptic activity and abnormal gene expression (Haydar and Reeves, 2012), highlighting the pleiotropic effects of this complex disease.

## Disclosure

The authors declare no conflicts of interest.

## Funding Support

The authors were supported by the CRTD, the TUD and the DFG Collaborative Research Center SFB655 (subprojects A20).
